# MicroRNA silencing: A promising therapy for Alzheimer’s disease

**DOI:** 10.46439/neuroscience.1.004

**Published:** 2020

**Authors:** Neelima B. Chauhan

**Affiliations:** Department of Pharmaceutical Sciences, School of Pharmacy, American University of Health Sciences, Signal Hill, CA 90755, United States

**Keywords:** Alzheimer’s disease, MicroRNA, AntagomiRs, Neuroinflammation

## Abstract

Alzheimer’s disease (AD) is a global health crisis currently afflicting ~6 million Americans (and ~40 million people worldwide). By the middle of the century, these numbers will stagger by ~16 million Americans (and ~152 million people worldwide) suffering from AD, if breakthrough disease-modifying treatments are not discovered. Currently, there are no treatments to prevent, halt or cure the disease. Multiple independent studies on brain gene expression patterns have indicated that in AD about 1/3^rd^ of the genes are upregulated while the rest 2/3^rd^ of the genes are downregulated. In that regard, AD therapeutics focused on antagomiR-mediated silencing of“upregulated”microRNAs (miRs) may be more feasible since upregulated miRs in AD continue to increase with the disease progression, as opposed to agomiR-mediated overexpression of down-regulated miRs with unpredictable reduced presence and relative short-life of 1–3h under pathological conditions in AD brain. Studies reported thus far indicate that most of the upregulated pathogenic genes in AD are regulated by pro-inflammatory microRNAs (miRs). Given the precedence of chronic neuroinflammation in triggering AD-like neurodegeneration and multifactorial nature of AD, silencing inflammation-specific micro-RNAs using antisense-microRNAs may be an effective adjuvant therapeutic strategy to prevent, halt or cure AD.

## Introduction

Alzheimer’s disease (AD) is a global health crisis currently afflicting ~6 million Americans (and ~40 million people worldwide). By the middle of the century, these numbers will escalate to ~16 million Americans (and ~152 million people worldwide) with AD, if breakthrough disease-modifying treatments are not discovered [1]. Currently, there are no treatments to prevent, halt or cure the disease. The FDA-approved symptomatic pharmacotherapy for AD include acetylcholinesterase inhibitors (AChEi) (Rivastigmine, Donepezil, Galantamine) and N-methyl-D-aspartate (NMDA) glutamate receptor modulator (Memantine) [2]. Investigational therapies for AD include antihypertensive drugs, anti-inflammatory drugs, secretase inhibitors, anti-diabetic insulin resistance drugs, brain-derived neurotrophic factor (BDNF), and immunization. Nutritional and botanical therapies include huperzine A, polyphenols, ginkgo, *panax ginseng*, *Withania somnifera* (Ashwagandha), phosphatidylserine, alpha-lipoic acid, omega-3 fatty acids, acetyl L-carnitine, coenzyme Q10, and melatonin, along with various vitamins and minerals. Other alternatives include physical exercise, cognitive training, leisure activities and socialization [3]. FDA-approved symptomatic pharmacotherapy provides only modest benefits without halting the progression of the disease and is associated with adverse effects [2,4,5] while other investigational therapies are not conclusive [3]. Given the lack of effective disease-modifying treatment(s) for AD [6], there is an unmet medical need in validating effective treatments with least/no side effects for treating AD [4,6]. There is a growing consensus that Alzheimer’s is a multifactorial disease involving a dysregulated interplay of multiple “aging” factors occurring much earlier than the actual onset of the disease [7], among which oxidative damage [8–10] constitutes one of the prime factors resulting from high energy requirement of brain with its modest anti-oxidant defense, and hence vulnerable to oxidative damage caused by reactive oxygen species (ROS) [11], along with chronic inflammation [12–14], cholinergic dysfunction [15–17], insulin resistance [18–20] and other factors. Recently, it has been implicated that increase in cerebral ß-amyloid (Aß) in the aging brain either due to reduced Aß clearance, influx of peripheral Aß resulting from age-dependent blood brain barrier (BBB)/blood cerebrospinal fluid barrier (BCSFB) breach, age-related oxidative damage and inflammation-PKR (Protein kinase RNA activated protein) induced Aß production [21], or Aß overproduction due to familial mutations, all leading to cerebral Aß accumulation that tend to destroy synaptic integrity fundamental to cognitive decline observed in prodromal AD and/or mild cognitive impairment (MCI) [7]. Oxidative stress and chronic neuroinflammation constitute the earliest biochemical changes triggering AD [11]. Emerging evidence indicates that these early biochemical changes in AD, are regulated by small non-coding microRNAs (miR/MiRs) [22]. MiRs are highly conserved ~22-nucleotide (nt) long non-coding RNAs that function as post-transcriptional regulators of gene expression shaping the transcriptome of a cell [23,24]. MiRs regulate gene expression by interfering with translation of their target messenger RNAs (mRNAs) via binding to the 3’-untranslated regions (UTR) of mRNAs to induce repression or degradation of target mRNA [25], thus blocking translation of target mRNA(s) into specific proteins [26,27]. Increasing evidence indicates crucial role played by miRs in human health and diseases [28,29]. There are about >3000 mature miRs currently characterized in human brain, of which only ~50 miRs have been found to be enriched within selective anatomical compartment(s) of the brain [23]. Increasing number of studies indicate that the dysregulation of miRs is fundamental to the etiology of neurodegenerative diseases including AD [7,30]. Multiple independent studies on brain gene expression patterns have indicated that in AD, about 1/3^rd^ of the genes are upregulated while the rest 2/3^rd^ of the genes are downregulated [31]. Interestingly, most of the upregulated pathogenic genes in AD are known to be under the transcriptional control of a pro-inflammatory mediators [31]. Inflammation-inducible miRs are found be significantly upregulated in AD-specific anatomic brain regions [32].

Early upregulation of neuroinflammation in AD and its persistence during the disease process in AD is characterized with the upregulation of NFkB as p50/p65 complex that controls diverse biological functions [33]. NFkB activation and binding to the promoters of NFkB-sensitive genes via miRs, facilitates transcriptions of many pathogenic genes altered in different neurodegenerative conditions including AD [32]. MiRs conventionally bind to the complementary RNA sequences in the 3’-UTR on mRNA and thereby repress the expression of target mRNA [32]. NFkB regulated miRs have been shown to be significantly elevated in AD brain, among which common to aging brain and AD brain i.e. miR-125b and miR-146a, are significantly upregulated [32]. Bioinformatics and multiple analytical techniques including RT-PCR, DNA-Array, Western blots, etc. have confirmed that both miR-125b and miR-146a target the 3’-UTR of several AD-related mRNAs [31,32]. MiR-125b was first shown to be upregulated in both stressed and differentiating mouse and human neurons, and has been implicated in neuronal development, cell-signaling and neurodegeneration [34]. NFkB-regulated miR-125b has been shown to be induced by neurotoxic aluminum sulfate that generates oxidative stress and ROS in human brain cells [35]. Consistent upregulation of miR-125b is associated with astrogliosis in various neurodegenerative conditions including AD [36]. MiR-125b is known to regulate neuronal synaptic functions, synaptic vesicle trafficking and neurotransmitter release, which when impaired in conditions such as AD, is reported to impair synaptic signaling and neurotransmitter release [37]. In addition, miR-125b is known to regulate cell cycle arrest and arachidonate 15-lipoxygenase (ALOX15) essential for conversion of docosahexaenoic acid (DHA) to neuroprotection D1 (NPD1), and therefore dysregulation of miR-125b leads to the down-regulation of cell cycle control and deficits in neurotrophic omega-3 fatty acids in the brain which in turn upregulates ß-secretase, prevents neurotrophic cleavage of ß-amyloid precursor protein (ßAPP) and increases Aß production [38].

On the other hand, miR-146a regulates complement factor H (CFH) and inactivates innate immune response in the brain, hence dysregulation of miR-146a leads to deficits in innate immune control and its chronic stimulation leading to pro-inflammatory signaling [39,40]. Additionally, miR-146a regulates interleukin-1 receptor-associated kinase 1 (IRAK-1), initiates innate immune response and activates NFkB signaling [39]. Moreover, miR-146a regulates transmembrane 4 superfamily member 12 (a regulator of cell surface receptor signal transduction), activates ADAM10-dependent neurotrophic cleavage of ßAPP, therefore when dysregulated, results in a shift from non-amyloidogenic to amyloidogenic processing of ßAPP [39]. In addition to the regulation of innate immunity, studies have shown that miR-146a also affects other biological functions such as hematopoiesis and cell differentiation [41]. Recently, Mai et al. have reported that although mir-146a was upregulated in hippocampus and temporal cortical brain regions critically involved in AD, its expression was unchanged in unaffected areas of AD brain, and showed that intranasal administration of miR-146a agomiR rescued pathological process and improved cognition in a mouse model of AD [42]. Another study by Salta et al. have shown that miR-132 involved in various aspects of central nervous system (CNS) functions, exhibited robust and consistent down-regulation in AD and that the therapeutic use of agomiR to miR-132 has a great potential in restoring AD-like pathogenesis [42]. Considering the therapeutic use of miRs in AD, it is logical to avoid overexpression of down-regulated miRs since for the most part, their down-regulation may be relevant to the relative short-life to the maximum of ~3h in AD brain and their reduced presence under pathological conditions which may promote rapid degradation [31,43]. Therefore, focusing AD therapeutics with the consideration of “upregulated” miRs may be more justifiable since upregulated miRs in AD continue to be upregulated with disease progression. Therefore, compared to agomiR-mediated overexpression of down-regulated miRs, antagomiR-mediated silencing of upregulated miRs may hold better therapeutic promise in treating AD.

### Anti-microRNA (AntagomiR) Strategies

The use of antisense complementary ribonucleotide sequences against the sequences of miR of interest (AntagomiRs) to lower the abundance of upregulated miRs for neutralizing down-stream pathogenic gene expression is coined as “AntagomiR strategy”. The activity of any given miR can be experimentally inhibited by an antisense oligos using locked nucleic acid (LNA), also known as 2’-O-Methyl nucleic acid (2MOE) modifications [44]. Of all, the LNA/2MOE RNA modification results in enhanced hybridization to target mRNA with increased sensitivity/selectivity/accuracy [31,45–47], traditionally confirmed by quantitative reverse transcriptase polymerase chain reaction (Q-RTPCR) quantitation of selected miR/mRNA [48]. Additional stability is attained by incorporating phosphorothioate linkage at the 5’-start and 3’-end nucleotides and cholesterol linkage to 3’-prime end, adds to the accuracy and reliability to LNA/2MOE modifications [47,49–51].

AntagomiRs have been successfully validated for their silencing efficacy of target mRNA in various *in vitro* and i*n vivo* systems [32]. PubMed literature search showed only four *in vivo* reports on the use of antagomiRs. Lee et al. showed that intraventricular administration of antagomiR-206 into the third ventricle of Tg2576 AD-mice, prevented detrimental effects of Aß42 on brain-derived neurotrophic factor (BDNF) cerebral levels and dendritic spine degeneration in neurons. Intraventricular delivery of AntagomiR-206 in Tg2576 mice increased BDNF levels, improved memory function, enhanced hippocampal synaptic density and neurogenesis [52]. These authors also attempted in parallel, intranasal administration of antagomiR-206 as a non-invasive delivery, which was found to reach the brain, increased cerebral levels of BDNF and improved memory function [52]. Another study by Zhang et al. showed that miR-299–5p treatment resulted in the attenuation of autophagy protein 5 (Atg5) antagonizing caspase-dependent apoptosis in the primary neurons from APPswe/PS1dE9 mice, N2a cells and SH-SY5Y cells, whereas antagomiR-299–5p enhance autophagy [53]. Another report by Wang et al. showed that intra-hippocampal delivery of antagomiR-146a into 5XFAD mice showed enhanced hippocampal levels of rho-associated coiled-coil containing protein kinase 1 (ROCK1) protein and repressed tau phosphorylation, partly restoring memory function in 5XFAD mice [54]. Lastly, Zhang et al. showed that overexpression of miR-214–3p in primary neurons from SAMP8 mice inhibited autophagy, and conversely, antagomiR-214–3p promoted autophagy and apoptosis in neurons from SAMP8 mice [55]. These reported studies indicate utilization of different routes of administration of miRs. Although intracranial, intrathecal, and intraventricular routes have an advantage of direct delivery of neurotherapeutics to the brain, the invasive nature of these routes poses practical limitations [56]. In that regard, intranasal route of drug delivery has gained considerable interest by virtue of being a safe, non-invasive route of administration that has proven efficiency of brain targeting [2,42,57–61].

Despite reported beneficial effects of various antagomiRs used in different *in vitro* and *in vivo* systems, the association of possible undesirable down-stream effects of miRs cannot be ruled out. As mentioned earlier, there are about >3000 mature miRs currently characterized in human brain, of which only selected ~50 miRs have been found to be enriched the brain [23] that have a potential to regulate a transcriptome of ~22,000–27,000 mRNAs [47]. Most miRs exhibit tightly regulated tissue- and cell-specific expression pattern and have an ability to potentially target the mRNA encoded by multiple genes simultaneously [31,60,62]. Even then, with a targeted delivery to the brain and advanced modifications of antagomiRs including 3’ adenylation, LNA modification with bicyclic furanose unit locked in an RNA-mimicking sugar conformation or other RNA chemical modifications increase the stability and specificity, and complementary binding 3’-UTR mRNA-miR ensures specificity [47]. Moreover, custom tailoring of short half-life of 3’-UTR mRNA-miR hybrid, significantly reduces the possibility of miR off-target undesirable effects [47]. Nevertheless, given the multifactorial heterogeneity of AD, therapeutic interventions for AD should be more of a combinational nature that would not only include RNA interference but also pharmacotherapy, alternative therapy and/or lifestyle/dietary changes [47].

## Conclusion

The use of antagomiR silencing therapeutic strategies in human neurological diseases such as Alzheimer’s disease are emerging. Advanced designing of miRs towards specific hybridization-affinity to targeted miR/mRNA, LNA nucleotide technology and nuclease resistance, along with relevant chemical modifications such as phosphorothioate backbone and cholesterol moiety inclusion have shown limited but proven success with least side effects under both *in vitro* and *in vivo* conditions. Considering the multifactorial nature of AD, therapeutic interventions of a combinational nature including tailored antagomiR strategies along with effective pharmacotherapy may ensure successful clinical outcome for Alzheimer’s cure.
